# Maxillary Antroliths: A Digital Panoramic-based Study

**DOI:** 10.7759/cureus.6686

**Published:** 2020-01-17

**Authors:** Georges Aoun, Ibrahim Nasseh

**Affiliations:** 1 Oral Medicine and Maxillofacial Radiology, Faculty of Dental Medicine, Lebanese University, Beirut, LBN

**Keywords:** lebanese, maxillary antrolith, panoramic, radiography

## Abstract

Objectives

Maxillary antroliths are calcified masses found within the maxillary sinus. The aim of this study was to investigate their presence in a sample of Lebanese population by means of digital panoramic radiographs.

Material and methods

In this study, 500 digital panoramic radiographs of Lebanese adult patients (281 females and 219 males) with a median age of 47.9±18.98 years were included and examined for maxillary antroliths. The statistical analysis of the data found was performed by IBM® SPSS® (IBM, Armonk, NY) version 20.0 for Windows.

Results

The sample investigated presented only three maxillary antroliths (0.6%). Among these, two were found in female patients and one in male; none of them was bilateral, one on the right side and two on the left side. No statistically significant relationships were observed with patients’ gender and age.

Conclusion

Maxillary antroliths are rare entities detected accidentally on panoramic radiographs utilized frequently in dental clinics. In light of their possible association with chronic sinusitis, dentists should have a complete knowledge of their diagnosis.

## Introduction

Maxillary antroliths (MAs) are calcified masses found in the maxillary sinus and result from the deposition of mineral salt around a foreign body (exogenous origin) or stagnant mucus (endogenous origin) [1,2]. Generally asymptomatic, MAs are rare entities detected accidentally on radiographic examinations (panoramic radiographs, computed tomography [CT], cone beam computed tomography [CBCT], etc.) [1-4]. In some cases, clinical symptoms such as facial pain, purulent and/or blood discharge, buccoantral communication, and others may be present [2,5-7]. Moreover, chronic sinusitis may result from infection around MAs [2,3,7]. Adversely, other studies suggest that chronic sinusitis can be behind the development of MAs as chronic infection can promote the deposition of mineral salts in the affected region [2,8]. Furthermore, MAs have also been found in sinuses infected by Aspergillus fumigatus [3].

Radiographically, MAs are described as faintly to extremely radiopaque masses embedded within the maxillary sinus. They are irregular, well-defined, and of different sizes and forms [2,3,7]. Their radiological differential diagnosis may include radiopacities located in the maxillary sinus such as residual root fragments, osteogenic (osteomas) and odotogenic lesions (cementoma), periapical condensing osteitis, and calcified neoplasms [4].

Normally, histology of MAs shows concentrical rings similar to those found in calculi located in different organs of the body covered by a granulation tissue richly vascularized. Chemical analysis of these entities highlights their formation of calcium phosphate, calcium carbonate, calcium oxalate, albumin, magnesium phosphate, organic substance, and water. As for their consistency, it can vary from hard and friable to soft, spongy, or crumbly. Finally, MAs can be of different colors (white, brown, gray, or black) [8].

With the absence of any radiological investigation for MAs in the Lebanese population, the aim of this study was to assess its prevalence in a sample of Lebanese population by means of digital panoramic radiographs.

## Materials and methods

In this retrospective study, digital panoramic radiographs of Lebanese adult patients were investigated. These radiographs, originally taken for dental reasons, were obtained from the archive of a radiology center located in Beirut. 

According to the center regulation, all patients gave their consent for future anonymous use of their radiographs for research purpose. The radiographs of patients who refused to consent were not added to the research archive.

All panoramic radiographs were obtained using the Pax Zenith digital panoramic unit (Vatech, Korea). The settings of the x-ray unit were chosen according to the patient profile (60-90 kV, 6-10 mA) with an exposure time between 10 and 20 seconds.

The exclusion criteria included the absence of patients’ information (age and sex) and low-quality images. 

Eventually, the sample was composed of 500 panoramic radiographs (219 males and 281 females) with an average age of 47.9 years.

One maxillofacial radiologist having more than 25 years of experience reviewed the radiographs for MAs; he used the same computer monitor and configuration to interpret the total sample.

Data collection took 10 sessions spaced by a 20-day period.

To avoid errors, 50 radiographs randomly chosen were rechecked 10 days after the first assessment while blinded to the primary results.

**Figure 1:**
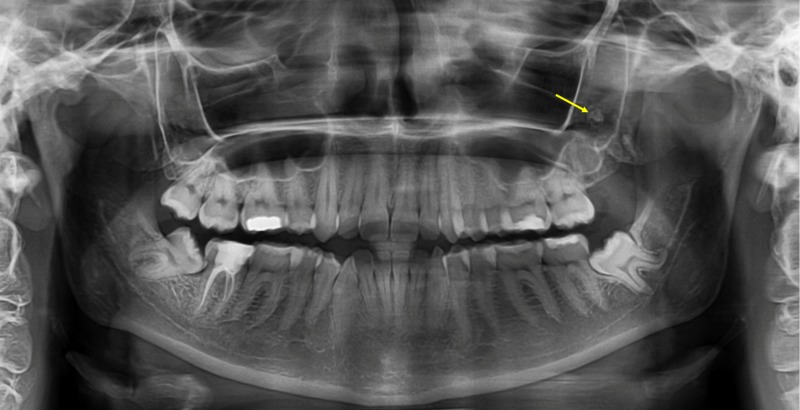
Antrolith within the left maxillary sinus A panoramic radiograph showing the presence of a maxillary antrolith within the left maxillary sinus.

**Figure 2:**
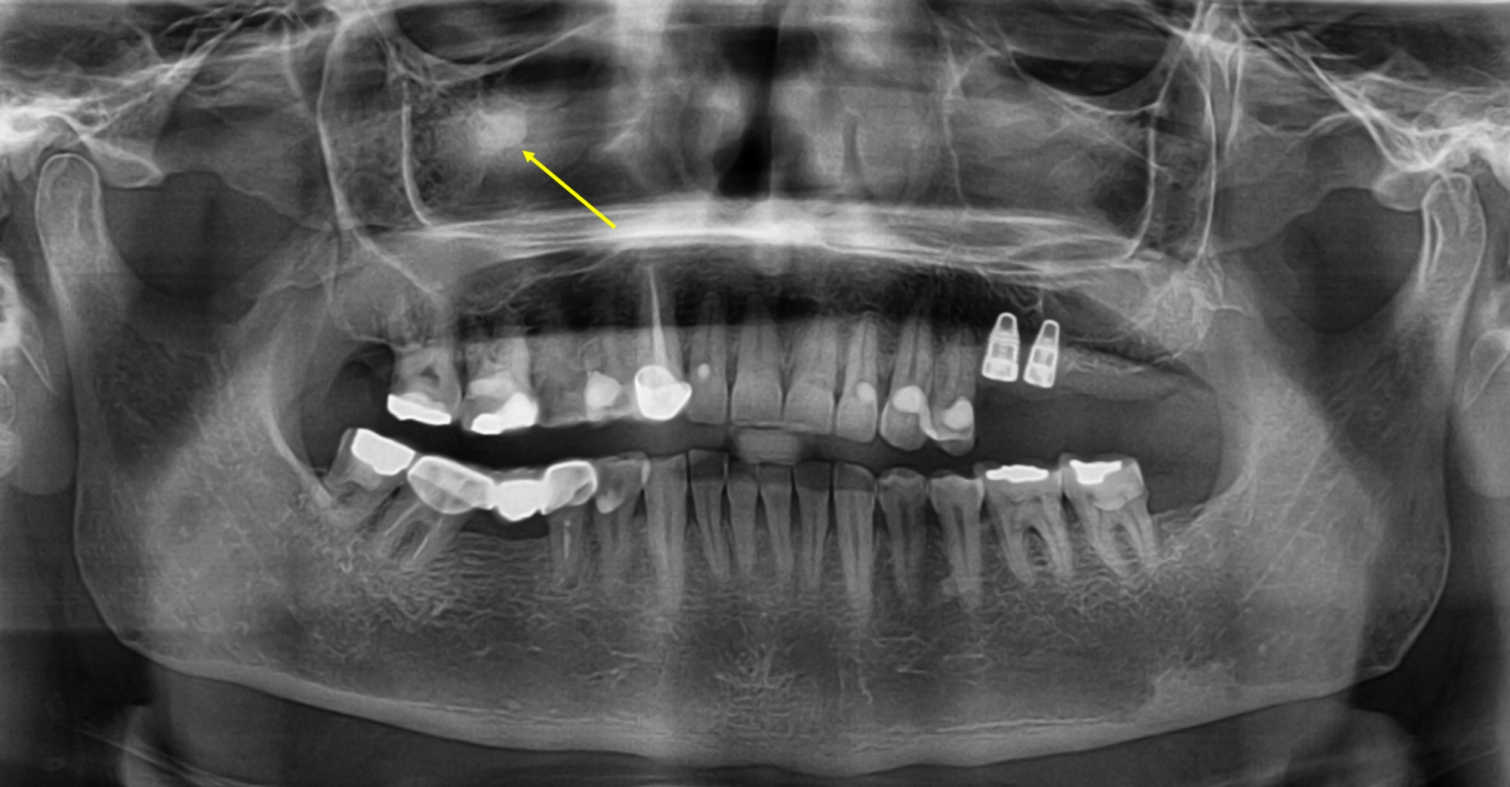
Antrolith within the right maxillary sinus A panoramic radiograph showing the presence of a maxillary antrolith within the right maxillary sinus.

MAs were identified as solitary or multiple radiopaque masses embedded within the antrum of the maxillary sinus, most frequently above the floor (Figures 1, 2).

Statistical analyses were carried out by IBM SPSS for Windows version 20.0 (IBM, Armonk, NY). Descriptive statistics of age, sex, and MAs were calculated and presented as frequency and percentage for categorical variables and as mean±standard deviation for continuous ones. Normality was evaluated for all the variables. Fisher’s exact test and Spearman correlation analysis were used to test statistical significance. P-values <0.05 were considered statistically significant.

## Results

In this study, the sample consisted of 500 adult patients (219 males, 43.8% and 281 females, 56.2%) aged 18-88 years (mean: 47.9±18.98 years).

MAs were seen in 0.6% (three cases) of the study population. Among these 0.2% (one case) was on the right side, 0.4% (two cases) on the left side (Table 1).

**Table 1:** Distribution of maxillary antroliths by side

	N	Percentage
Right side only	1	0.2
Left side only	2	0.4
Bilateral	-	-
None	497	99.4
Total	500	100

As for the descriptive statistics based on sex, MAs were seen in:

- 0.7% (two cases) of the female study population, one (0.35%) on each side.

- 0.45% (one case) of the male study population, on the left side (Table 2).

**Table 2:** Distribution of maxillary antroliths by sex

	Presence of maxillary antroliths	Percentage
Male (n=219)	1	0.45
Female (n=281)	2	0.7

Fisher’s exact test revealed insignificant association between gender and MAs (p=1>0.05).

The mean age for patients affected by MAs was 40.6 years (ranges from 23 to 53 years). Spearman correlation analysis showed a non-significant, very weak negative correlation between age and MAs (p=0.499>0.05).

These statistical results were expected due to the small number of cases obtained in this study.

## Discussion

MAs are calcifications that form within the maxillary sinus as a consequence of mineral salt deposition around an exogenous (piece of cotton, dental implants, gutta-percha cones, etc.) or endogenous (tooth, osseous fragments, mucus, etc.) nucleus [3,9-14]. This formation process is not yet clear, but chronic infections (bacterial and fungal), reduced sinus drainage, and the existence of foreign bodies are risk factors [9,11].

Panoramic radiography is frequently requested by dentists in daily practice. It provides essential information that is difficult to obtain from clinical assessment such as horizontal alveolar bone loss, dental impaction patterns, periapical lesions, and dentoalveolar fractures [15]. Additionally, it is known to be an effective technique in the detection of some maxillary sinus pathologies.

In the present study, we investigated MAs in a sample of adult Lebanese patients by means of digital panoramic radiographs. In the literature, a very small number of studies explored MAs using this radiological technique; the majority used CBCT in their MAs assessment.

In our study, the prevalence of MAs was 0.6% (three cases out of 500 images reviewed). Interestingly, in their study, Nass Duce et al. found the same number of cases (three) but in a larger sample (1,957 radiographs) taken originally with a different imaging technique (paranasal sinus CT); their incidence of MAs was 0.15% [16].

On the other hand, other studies reported slightly higher occurrences of MAs than ours (Cho et al. 0.99%, N=13,946, CBCT technique; Lana et al. 3.2%, N=500, CBCT technique; Rege et al. 3.2%, N=1,113, CBCT technique) [1,17,18]. This small variation existing between all these studies and ours might be related to the assessed images. In fact, because of the anatomic structures superimpositions caused by the two-dimensional radiography, panoramic technique could miss small MAs. In contrast, CT and CBCT can undoubtedly detect MAs independent of their size and shape. However, the cost and the high radiation dosage of CT limit its use in favor of 2D and CBCT techniques, which can offer helpful information on MAs without high radiation exposure [19].

Finally, our study aiming to estimate the incidence of MAs in an adult Lebanese population is not without limitations. A bigger sample and the evaluation on 3D images can, definitely, lead to more precise results.

## Conclusions

MAs are rare calcifications located within the maxillary sinus. In daily dental practice, they could be accidentally discovered on digital panoramic radiographs taken for diagnostic purposes. Dentists should be aware about these entities in order to prevent subsequent complications mainly chronic sinusitis.
